# Effects of happy and angry human voice recordings on postural stability in dogs: An exploratory biomechanical analysis

**DOI:** 10.1371/journal.pone.0339979

**Published:** 2026-01-28

**Authors:** Nadja Affenzeller, Masoud Aghapour, Christiane Lutonsky, Christian Peham, Barbara Bockstahler

**Affiliations:** 1 Clinical Department for Small Animals and Horses, Clinical Centre for Small Animal Health and Research, Physical Therapy, University of Veterinary Medicine (Vetmeduni), Vienna, Austria; 2 Clinical Department for Small Animals and Horses, Clinical Centre for Small Animal Health and Research, Behavioural Medicine, University of Veterinary Medicine (Vetmeduni), Vienna, Austria; 3 Clinical Department for Small Animals and Horses, Clinical Centre for Equine Health and Research, Movement Science Group, University of Veterinary Medicine (Vetmeduni), Vienna, Austria; Universita Politecnica delle Marche Facolta di Ingegneria, ITALY

## Abstract

Auditory stimuli are known to induce biomechanical balance responses, influencing postural stability in humans. These responses provide valuable insights into the interaction between auditory perception and physical balance. This study investigates the effect of human voices on postural stability in dogs during static stance. Twenty-three healthy pet dogs were assessed standing on a pressure plate under three auditory conditions: happy voice, angry voice, and no sound. Five conventional Center of Pressure (COP) parameters were analyzed, mediolateral displacement, craniocaudal displacement, support surface (SS_%), average speed (AS) and statokinesiogram length. A significant main effect of condition on SS_% (*F* (2) = 4.35, p = 0.019, η²_p_ = 0.165) was found; SS_% values in the angry voice condition (mean = 0.12 ± 0.06) were significantly higher than in the no sound condition (mean = 0.08 ± 0.03; p = 0.026). A K-means cluster analysis of relative COP changes (ΔCOP_%, increase/decrease relative to the no sound condition) revealed two distinct reaction patterns within both sound conditions (ANOVA, all ΔCOP_% parameters, p < 0.01, except AS within happy condition). For happy voices, 57% of dogs exhibited increases across all ΔCOP_% parameters, while 43% showed decreases. In contrast, angry voices led to increased ΔCOP_% parameters in 30% of dogs, with 70% remaining unaffected. A significant difference in Support Surface (ΔSS_%) was found between clusters 1 for happy and angry voices (F = 8.75, p = 0.008). The largest absolute and relative ΔSS_% changes occurred in the angry voice condition. These exploratory findings suggest that the emotional arousal triggered by human voices can have both stabilizing and destabilizing effects on canine balance. Angry human voices were associated with the greatest destabilizing effect.

## Introduction

Postural stability is a fundamental motor function that enables humans and animals to maintain stability and prevent falls. Balance is sustained through coordinated muscle contractions acting against gravity, forming the foundation for activities such as walking and standing. Even standing still is an active, controlled process, governed by the integration of sensorimotor signals [1,2]. While visual, proprioceptive, and vestibular inputs are traditionally recognized as primary contributors to balance control, recent studies in humans have revealed the significant role of external auditory stimuli in modulating postural stability [3].

Human research suggests that the effects of auditory stimuli on balance are context dependent. High-frequency sounds, for instance, have been shown to disrupt postural stability [4], whereas auditory conditions including white noise may enhance balance [5]. A recent meta-analysis reported that auditory cues can improve postural control, but the high variability across studies highlights the complexity of this relationship [3]. Additionally, emotional stimuli have been linked to postural changes, with studies in humans demonstrating balance shifts in response to pleasant and unpleasant sounds, often interpreted as approach-withdrawal behaviors. High-arousal affective stimuli have also been associated with freezing responses [6].

These findings underscore a dual role for sound as both a stabilizing and destabilizing factor, depending on the context and the emotional content of the auditory stimuli.

While the influence of auditory stimuli on balance has been extensively studied in humans, there is limited research on how sound affects balance in animals, including dogs. Canine studies have primarily focused on behavioral responses to specific sounds, such as fireworks [7], leaving a gap in understanding the biomechanical effects of auditory stimuli on postural control. Standardized methods like posturography offer a robust tool for evaluating postural control by analyzing the displacement of the center of pressure (COP) within the base of support (BOS) of a subject [6,8]. BOS is the area outlined by the contact points between the body and the supporting surface [8]. Posturographic assessments during gait (dynamic) or quiet stance (static) rely on recording ground reaction forces (GRF) [9,10]. The vertical GRF represent the sum of all vertical forces between a physical object and its contact surface. The center of pressure (COP) corresponds to the point where the instantaneous GRF vector is applied [1]. COP displacement serves as an indirect indicator of postural control performance and, by extension, of balance maintenance ability [11]. Conventional COP metrics, including anterior-posterior (craniocaudal in dogs) and mediolateral displacement, total length of the COP excursion, average speed, and support surface, provide indirect measures of balance stability [1,12,13].

This study aims to explore the impact of emotional auditory stimuli on canine balance using posturographic analysis. Specifically, we investigate how recordings of happy and angry human voices influence postural stability in dogs during static stance. By examining 5 COP parameters across 3 auditory conditions (happy voice, angry voice, and no sound), this research seeks to provide insights into the interplay between emotional auditory cues and biomechanical balance responses in dogs.

## Materials and methods

### Approval and consent

This study received approval from the Ethics and Animal Welfare Committee at Vetmeduni, University of Veterinary Medicine, in accordance with institutional policies for Good Scientific Practice and applicable national regulations (ETK-148/10/2021). Written informed consent was obtained from all dog caretakers prior to participation.

The human voice recordings used in the study were provided by Natalia Albuquerque [14]. Written informed consent was obtained from the individuals whose vocalizations were recorded, including consent for use in research and related publications. All procedures were carried out in accordance with relevant ethical standards and regulatory guidelines. Ethical approval was granted by the Ethics Committee of the School of Life Sciences, University of Lincoln, UK [14].

### Animals

A total of 27 client-owned healthy dogs were evaluated in this study. The inclusion criteria required the absence of clinical musculoskeletal, neurological, or visual disease, and all dogs underwent a comprehensive clinical examination by qualified veterinarians including visual gait assessment, orthopedic evaluation, and calculation of symmetry index (SI, for details see data analysis) when walking. Four dogs were excluded due to their inability to remain still while exposed to the voice recordings. The dog breeds included mixed breeds (n = 5), Labrador retriever (n = 5), border collie (n = 5), Golden retriever (n = 2), standard poodle, Malinois, Irish terrier, pointer, greyster and Austrian pinscher. The dogs’ body mass ranged from 13.5 to 30.6 kg (22.3 ± 5.6), and their ages ranged from 1.4 to 6.3 years (3.6 ± 1.4). Thirteen males (10 intact, 3 neutered) and 10 females (4 intact, 6 spayed) participated. All dogs were required to have more than 10 kg body mass. All caretakers have completed basic obedience training with their dogs, including a “wait in standing position” command.

### Equipment and measurement procedure

**Fig 1:**
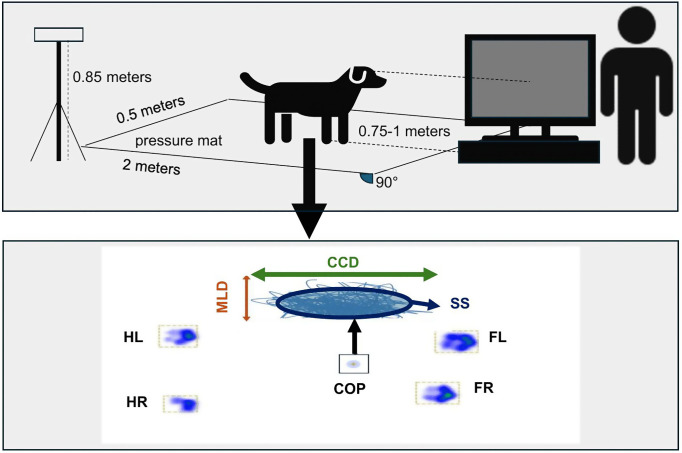
Schematic set up. The dog is positioned on top of the pressure mat facing the screen. The dog handler is standing behind the screen. The camera is placed on top of a tripod and records each trial. CCD: craniocaudal displacement of the COP; COP: Center of Pressure; FL: left front leg; FR: right front leg; HL: left hind leg, HR: right hind leg; MLD: mediolateral displacement of the COP; SS: Support surface; blue thin line: statokinesiogram, path of the COP; graphic adapted from [11].

In this prospective study, the COP parameters of the dogs were measured in a quiet standing position on a flat ground by using a Zebris platform (FDM Type 2, Zebris Medical GmbH, Allgäu, Germany) equipped with 15, 360 sensors covering an area of 203 × 54.2 cm and a measuring frequency of 100 Hz. The sensor size of the platform was 0.72 × 0.72 cm. To standardize the coefficient of friction, the pressure plate was covered with a black 1-mm-thick non-slip rubber mat made of polyvinyl chloride. All measurement procedures were filmed using a Panasonic NV-MX500 camera (Panasonic, Kadoma, Osaka, Japan) with a standardized setup for camera positioning and angle. The camera was positioned to capture the dog standing in front of a TV screen while the auditory stimuli were played over the TV speakers (Samsung, UE40B6000, Samsung Electronics Co., Ltd., Suwon, South Korea). A schematic set up is shown in Fig 1. The screen displayed a black image with the condition name (i.e., man angry, written in white color) in the upper left corner. Speaker volume was kept constant for all dogs.

The auditory stimuli consisted of recordings of 2 humans (1 female, 1 male) speaking in an unfamiliar language expressing either positive (happy) or negative (angry) emotions. The recorded words were the novel exclamation “venha cá” (“come here!” in Brazilian Portuguese) said in a happy or angry tone performed by adult drama students. All recordings were generated in a soundproof room in mono with a sampling rate of 48kHz using Raven Pro 1.4 software (www.birds.cornell.edu) and 5s sound clips for each vocalisation type were generated and controlled for average volume (54.7 dB). These recordings have previously been used to demonstrate the ability of pet dogs to recognize human emotions and were generously provided by Albuquerque and her colleagues [14]. The stimuli were presented in a pseudorandomized order featuring both the happy and the angry voice from the female and the male speaker. Each auditory stimulus was played once for 5 seconds per trial. Stimuli order in trial 1 was female happy, male angry, male happy, female angry, in trial 2 female angry, male happy, male angry, female happy and in trial 3 female happy, male happy, female angry and male angry. Therefore, each dog heard each possible combination (male, female, happy, angry) once per trial. Each dog took part in 3 trials, resulting in hearing the happy male voice 3 times, the happy female voice 3 times, the angry male voice 3 times, and the angry female voice 3 times.

### Static posturography

To familiarize the dogs with the room, the set-up, and the equipment, they were allowed to wander around freely for up to 15 minutes. Once the dogs were familiarized with the room, GRF were calculated when walking in a straight line using the pressure plate mentioned above. At least five valid passes were analyzed to calculate the SI for peak vertical force (PFz) and vertical impulse (IFz). A valid pass was defined as a walk in which the dog crossed the plate without changing pace, turning its head, pulling on the leash, or touching the dog handler. The difference in speed at which the dogs crossed the plate had to be within a range of ± 0.3 m/s and an acceleration of ± 0.5 m/s^2^. The SI for PFz, and IFz of all included dogs in this study was below 3% which is the margin typically used to diagnose a lameness free gait pattern [15].

All dogs were positioned facing the screen from approx. 0.5 − 0.75m away by using positive reinforcement methods. Then the dog handler walked behind the screen, turned around and faced the dog without any further interaction (for details and set up see Fig 1). When hearing the 3 different auditory conditions the dogs had to stand still on the pressure platform with all limbs perpendicular to the platform without receiving or seeing any treats or treat pouches. The dogs were able to have a break between trials and received treats after each trial. Data were discarded by the observer if any body, head, tail, limb, or paw movements were observed on the video recording.

### Data analysis

All data were analyzed with the custom-made software Pressure Analyzer (Michael Schwanda, version 4.8.7.0), and exported to Excel (Microsoft, Excel, 2016) for further analysis.

The following parameters were used for the evaluation of the inclusion criteria:

- The mean speed (m/s) and acceleration (m/s²) were calculated for the left forelimb.
- Symmetry index (SI) expressed as a percentage (SI%), was calculated for both parameters (PFz and IFz) according to the following equation:

SIXFz (%)=abs ([ XFzLLx – XFzRLx ] /[XFzLLx + XFzRLx]) x 100 

where XFz is the mean value of PFz or IFz of valid steps, LLx is the left front or hindlimb, and RLx is the right front or hind; perfect symmetry between the right and left front or hindlimbs was assigned a value of 0%.

For posturographic analysis, all data were low-pass filtered using a fourth-order Butterworth Filter with a cutoff frequency of 10 Hz [16,17]. The following parameters were analyzed:

- Base of support (BOS): area enclosed by the coordinates of the center of the paws in square centimeters (cm^2^).
- Base of support length (BOS L): distance between the center of the front and hindlimbs in centimeters (cm)
- Base of support width (BOS W): distance between the center of the left and right limbs in centimeters (cm).
- Craniocaudal displacement: Mean deviation on the craniocaudal axis in millimeters (mm). It was normalized to the BOS L and expressed as a percentage (CCD_%).
- Mediolateral displacement: Mean deviation on the lateral axis millimeters (mm). It was normalized to the BOS W and expressed as a percentage (MLD_%).
- Statokinesiogram length: the length of the line that joins the points of the COP trajectory in meters (m). It was normalized to the BOS and expressed as a percentage (L_%).
- Support surface or statokinesiogram: The area determined by an ellipse that contains 90% of the points of the COP trajectory in mm^2^. It was normalized to the BOS and expressed as a percentage (SS_%).
- Mean speed (AS) (mm/s) of COP sway.

Based on a validity study of static posturography measurements in dogs, the mean COP values from 2 valid trials for each auditory condition were calculated and used for further analysis for each dog, to reduce within subject variability and improve reliability [18].

For statistical analysis IBM SPSS version 30 (IBM, Chicago, USA) was used. Descriptive statistics were calculated for all COP data. Data was analyzed for normality assumption using the Shapiro-Wilk test. To determine significant differences between sound condition (no sound, happy, angry), a repeated measures ANOVA test was conducted for each dependent variable across the 3 conditions. Mauchly’s test of sphericity, Greenhouse–Geisser corrections where necessary, effect sizes (partial η²), and Bonferroni-adjusted pairwise comparisons were used. SS_% was defined a priori as the primary endpoint. To control multiplicity across secondary endpoints, p-values were adjusted using the Holm–Bonferroni procedure, implemented in SPSS by ranking p-values and applying sequential multiplicative corrections (familywise α = 0.05).

For each dog and all COP parameters individual reactions were calculated and expressed as percent increase or decrease where the COP values of the no sound condition served as baseline. Individual percentage reactions were calculated for both sound conditions (angry and happy) separately.

Individual percentage COP reactions were labeled with a Δ symbol and were expressed as ΔMLD_%, ΔCCD_%, ΔL_%, ΔAS, ΔSS_% using the following formula exemplarily shown for ΔMLD_%:


$$\Delta MLD\_\;\%=\frac{MLD\_\%\;(human\;sound\;condition)-MLD\_\%\;(no\;sound\;condition)}{MLD\_\%\;(no\;sound\;condition)}x\text{1}\text{0}\text{0}$$

To classify individual dog reactions cluster analyses were conducted, grouping all COP data based on their relative similarity. A two-stage analysis was performed. The initial step involved a Hierarchical cluster analysis using Ward’s method and squared Euclidian distances to explore the data structure. Based on the resulting dendrogram, a K-means cluster analysis was subsequently performed to partition the data into distinct clusters. An ANOVA was used to evaluate statistically significant differences in all COP parameters both within and between the identified clusters.

## Results

The means of all COP parameters of 2 valid trials per condition were analyzed for all 23 dogs included in this study (for details see Table S1); the mean duration of hearing the happy voice was 4.9 ± 0.1s, for the angry voice 4.8 ± 0.2s and for the no sound condition 5.0 ± 0.0s.

A repeated-measures ANOVA revealed a significant main effect of condition on SS_%, F (2) = 4.35, p = 0.019, η²_p_ = 0.165, indicating a large effect. Bonferroni-adjusted pairwise comparisons showed that SS_% values in the angry voice condition (mean = 0.12 ± 0.06; 95% confidence interval: 0.096, 0.144) were significantly higher than in the no sound condition (mean = 0.08 ± 0.03; 95% confidence interval: 0.068, 0.097; p = 0.026), whereas the difference between angry sound condition and happy sound condition was not significant (p = 1.000). Mauchly’s test showed sphericity was met (W = 0.981, χ²(2) = 0.41, p = 0.817).

For MLD_%, a significant main effect of condition was found, F (2) = 4.28, p = 0.02, η²_p_ = 0.163.

However, Bonferroni-corrected pairwise comparisons did not reveal any significant differences between individual conditions (all p > 0.05).

To control multiplicity SS_% was defined a priori as the primary endpoint. After Holm adjustment across the secondary endpoints, no other COP parameters were significant (MLD_% p- adjusted = 0.08; CCD_% p- adjusted = 0.83; AS p- adjusted = 1.00; L_% p- adjusted = 1.00). Descriptive statistics and detailed repeated measure ANOVA outcome are reported in Table 1 and Table S2.

**Table 1:** Descriptive statistics (mean values ± SD) of all 5 COP parameters in different auditory conditions and repeated measure ANOVA outcome.

Parameter	Angry	Happy	No sound	F	p-value (Holm-adj.p)	η²_p_	Effect size	Sign. pair. diff.
MLD_%	1.54 ± 0.43	1.50 ± 0.41	1.27 ± 0.32	4.28	0.02 (0.08)	0.163	large	all p > 0.05
CCD_%	1.27 ± 0.30	1.25 ± 0.29	1.17 ± 0.26	1.33	0.28 (0.83)	0.057	small	all p > 0.05
L_%	0.11 ± 0.05	0.11 ± 0.05	0.11 ± 0.05	0.07	0.89 (1.00)	0.003	small	all p > 0.05
AS	22.53 ± 8.72	22.00 ± 9.43	20.88 ± 7.15	0.48	0.55 (1.00)	0.021	small	all p > 0.05
SS_%	0.12 ± 0.06	0.11 ± 0.06	0.08 ± 0.03	4.35	**0.02**	0.165	large	yes (Angry > No sound)
								

MLD_%: mediolateral displacement; CCD_%: craniocaudal displacement; L_%: length of the COP; AS: average speed of the COP; SS_%: support surface; p-values are based on repeated-measures ANOVA, where Mauchly’s sphericity was violated, Greenhouse–Geisser corrections were applied; Holm-adj.p: Holm-adjusted p values; F: F-test value; η²_p_: partial Eta squared; effect size interpretation based on Cohen’s guideline: small < 0.06, medium = 0.06–0.14, large > 0.14); sign. pair. diff: significant pairwise differences; Angry: hearing angry human voice recording; Happy: hearing happy human voice recording; %: denotes parameters that were normalized to allow comparison across individuals based on their BOS data in each trial; bold values indicate significant results (p* *< 0.05).

When analyzing the individual percentage reactions of all dogs in all COP data large differences were observed. The smallest difference was ΔCCD_% and ranged from a decrease of −40.7% (dog Desper, happy voice) to an increase of 96.7% (dog Quentin, angry voice). The highest difference was ΔSS_% and ranged from −60.0% (dog Grisu, angry voice) to 228.6% (dog Hiska, angry voice). Details on individual dogs and their absolute and relative differences of all COP parameters including the cluster numbers are presented in supplements Table S1.

Inspection of the Hierarchical Cluster analysis dendrogram supported a 2-cluster solution. Subsequently, a *k*-means cluster analysis (*k = 2*) identified 2 distinct clusters in both sound conditions (happy, angry) (see Table 2). Box Plot graphs depicting all ΔCOP_% data between the clusters are presented in supplements S3 Fig (A-E).

**Table 2:** Results of K-means cluster analysis based on sound condition.

Cluster	Condition angry		ANOVA		Condition happy		ANOVA	
	1	2	F	p	1	2	F	p
ΔMLD_%	77.60	5.83	24.33	<0.001*	49.03	−9.04	22.342	<0.001*
ΔCCD_%	49.21	−2.27	23.12	<0.001*	27.12	−7.11	9.298	0.006*
ΔL_%	61.65	−8.40	13.40	0.001*	27.62	−19.23	11.337	0.003*
ΔAS	56.14	−2.45	11.02	0.003*	22.12	−6.97	3.663	0.069
ΔSS_%	165.88	16.96	54.92	<0.001*	99.68	−14.47	41.842	<0.001*
dogs	7	16			13	10		

cluster: cluster number (1, 2) based on K-Means cluster analysis; ΔMLD_%: relative mediolateral displacement (%); Δ_CCD %: relative craniocaudal displacement (%); ΔL_%: relative length of the COP (%); ΔAS: relative average speed of the COP (%); ΔSS_%: relative support surface (%); dogs: number of dogs within the cluster; Happy: hearing happy human voice recording; Angry: hearing angry human voice recording; %: denotes parameters that were normalized to allow comparison across individuals based on their BOS data in each trial; F: F-test value; *p < 0.05 statistically significant.

ANOVA analysis confirmed significant differences between both identified clusters in all ΔCOP_% parameters within both sound conditions (except for ΔAS, condition happy, p = 0.069).

When hearing the angry voices, dogs grouped in cluster 1 showed an overall increase in all 5 ΔCOP parameters (30% of dogs n = 7) whereas dogs grouped in cluster 2 (70% of dogs, n = 16) remained mostly stable (range of change from −8.4 to 16.96%). When hearing the happy voices, dogs grouped in cluster 1 (57% of dogs n = 13) showed an overall increase in all 5 ΔCOP parameters, whereas dogs grouped in cluster 2 (43% of dogs, n = 10) predominantly decreased in all 5 ΔCOP parameters (for details see Table 2).

When comparing all ΔCOP_% parameters of dogs based on their cluster number, ANOVA analysis confirmed a significant difference in ΔSS_% between both clusters 1 (F = 8.75; p = 0.008) and a non-significant difference in ΔSS_% between both clusters 2 (F = 3.92; p = 0.059).

## Discussion

Auditory stimuli are known to induce biomechanical balance responses, influencing postural stability in humans. This study explored the effects of emotional auditory stimuli, specifically happy and angry human voice recordings, on postural stability in pet dogs during static stance. The emotional arousal when hearing human voices had both a stabilizing and destabilizing effect on balance in dogs.

When analyzing the 3 auditory conditions a significant main effect of the angry voice condition on SS_% was found; SS_% values in the angry voice condition were significantly higher than in the no sound condition (p = 0.026). Support surface captures 90% of the two-dimensional overall direction of the COP excursion (in mediolateral and craniocaudal direction) typically forming an ellipse representing the sway area. Hence, this significantly increased SS_% when hearing angry voices indicates postural instability as the body exhibits greater movements to maintain balance [19]. This result aligns with previous findings indicating that high-arousal stimuli, particularly those associated with negative emotions, can destabilize postural balance [3,6].

Most interestingly when COP data was analyzed on an individual dog level, we found that individual reactions varied quite extensively. While COP values of some dogs considerably increased, they decreased in other dogs. This difference in response may also explain the non- significant results in the other 4 COP parameters when analyzing the 3 auditory conditions. This variability observed in ΔCOP_% responses highlight the complexity of auditory-emotional interactions, consistent with studies demonstrating that emotional responses and postural adjustments vary significantly and could be based on individual sensitivity and prior experiences [4,5]. Indeed, grouping the dogs using cluster analysis revealed that 13 of 27 dogs in the happy voice condition and 7 out 27 dogs in the angry voice condition reacted with an overall increase in all 5 ΔCOP_% parameters when hearing the voice recordings. This finding implies a destabilizing effect on a subpopulation of dogs on balance. While emotional valence (positive or negative) is often emphasized in studies of emotional stimuli, evidence increasingly suggests that arousal, rather than valence, is the key determinant of postural responses [20]. For example, although human studies have demonstrated that unpleasant visual stimuli frequently elicit freezing behaviors, other studies report an increase of COP parameters to similar stimuli [21–24]. These findings align with our observation that both happy and angry voices elicited increased ΔCOP_% in a subpopulation of dogs (clusters 1), suggesting that the arousal induced by the stimuli, rather than their valence, played a more significant role in shaping postural strategies.

Surprisingly, a more stable balance (an overall reduction in all ΔCOP_% parameters), often interpreted as freezing behavior to a threat, was observed when hearing happy voices in 10 dogs. None of the dogs displayed any behavioral signs indicative of distress or fear when hearing any of the voice recordings, which is why a perceived threat appears rather unlikely. Notably, freezing responses are not exclusive to negatively valenced stimuli; similar reactions have been observed in response to positive stimuli [25]. We propose that some of the observed postural responses, particularly in this cluster of dogs, may reflect anticipatory strategies- a phenomenon known from humans. Anticipatory postural adjustments (APAs) are defined as the activation of postural muscles in a feedforward manner to prepare for destabilizing forces associated with voluntary movement [26]. APAs are learned behaviors based on prior experiences of postural disturbances and serve to optimize stability during anticipated challenges [27]. Dogs that displayed predominantly decreased ΔCOP_% parameters may have engaged in preparatory postural adjustments when hearing happy voices. This could be indicative of a learned response to manage the anticipated impact of emotionally salient happy auditory cues, possibly influenced by the presence of their handler in the testing room or previous exposure to similar situations. Indeed, the stabilizing effects of happy voices in cluster 2 of dogs may reflect attentional focus in anticipation of a positive interaction with the dog handler who was standing in front of all dogs, rather than a behavioral freezing response in a perceived threat.

Nevertheless, it is important to point out that the most pronounced destabilizing effect was observed in 30% (7/23, cluster 2) of dogs when hearing angry voice recordings. While the smallest increase centered around 49% in craniocaudal direction (ΔCCD_%) the highest increase was observed in Support Surface (ΔSS_%) with 166%. As discussed earlier, negative stimuli likely triggered increased arousal, and the negative valence may have further influenced balance control. SS is a comprehensive indicator of postural stability, directly related to an individual’s ability to maintain their center of mass within the BOS. It is already known that SS changes depending on the complexity of the task, the surface conditions and the individual’s balance capability [28,29]. The exploratory findings of this study emphasize the effect of hearing human voice recordings as highly arousing stimuli in addition to the negative emotional valence of an angry voice in a subpopulation of dogs for balance control.

However, for the majority (70%) of dogs, angry voices did not notably affect balance. None of the dogs showed behavioral indicators of conflict or fear while participating in the study (determined by the first author NA who is a Diplomate of the European College for Animal Welfare and Behaviour Medicine); all of them were considered emotionally healthy. Dogs can identify emotions of humans when hearing voice recordings while also seeing corresponding human faces on a screen [14]. It can therefore be argued that the discrepancy between the recording of an angry voice and the presence of a neutral dog handler was not perceived as arousing or threatening by the dogs.

Several limiting factors must be considered when interpreting these findings. The substantial individual variability observed in ΔCOP_% responses highlight the need for larger sample sizes. Because the clustering procedure and subsequent statistical testing were based on the same COP variables, the analyses are exploratory and intend to describe rather than confirm potential subtypes of postural responses. Future work should assess cluster quality and stability and validate the cluster structure in an independent canine dataset.

In addition, consideration of factors such as life stages (age of dogs and their prior life experiences) and individual dog temperament might have affected data outcome. Although all dogs were considered emotionally healthy and no dog showed behavioral signs of stress, positive and negative individual prior life experiences may have affected postural responses. Additionally, various breeds with different ear morphologies (pricked ears and drooping ears) took part in the study. While there is no published research on breed-specific differences in sensory processing of human voices or data on how ear shape influences hearing ability, these factors cannot be ruled out as a potential limitation of the study.

Although care was taken to standardize the playback setup across all trials, absolute sound pressure level (SPL) values at the individual dog ear could not be determined. Performing individual in-ear or near-ear SPL calibration would have required placing a microphone close to the dog’s head, which could have interfered with the animal’s natural posture and behavior, making it even more difficult for the dogs to remain still. Therefore, all playback parameters (device type, output settings, distance to speaker 0.5m – 0.75m, and room conditions) were kept constant to minimize variability in sound exposure across individuals. We believe that this approach ensured comparability across conditions in this exploratory study, although absolute SPL values at the ear could not be determined.

Additionally, integrating electromyography data to capture muscle activity could provide more direct evidence for anticipatory adjustments behaviors in dogs. Incorporating heart rate and heart rate variability measures in future studies could help analyzing arousal and valence aspects. Finally, the sight of the dog handler may have influenced the dogs’ responses, an aspect that warrants further investigation.

In conclusion, this exploratory study in dogs provides preliminary evidence that emotional auditory stimuli may evoke diverse postural responses, shaped by the nature of the stimulus (happy or angry) and individual differences in emotional reactivity and sensory processing. The observed patterns suggest that these responses could be more closely related to arousal than to valence, with both positive and negative cues eliciting distinct postural adjustments. Angry human voices appeared to have the largest destabilizing effects, possibly reflecting heightened arousal alongside the negative valence of this stimuli in a subgroup of dogs.

On the contrary, increased stability in some dogs when hearing happy voices were attributed to anticipatory adjustments (rather than freezing in response to a threat). However, these findings should be interpreted with caution given the exploratory design and the study’s methodological limitations. We believe that these findings provide new insights into the interplay between auditory stimuli, emotional processing, and postural control in dogs, contributing to a growing understanding of sensory-emotional integration in non-human species.

## Supporting information

S1 Table Descriptive statistics of all COP parameters and cluster number in different auditory conditions for each individual dog. MLD_%: mediolateral displacement; CCD_%: craniocaudal displacement; L_%: length of the COP; AS: average speed of the COP; SS_%: support surface; Δ: individual dog reaction expressed as percent difference when compared to the no_sound condition for every COP parameter; cluster: cluster number of each dog based on cluster analysis; Happy: hearing happy human voice recording; Angry: hearing angry human voice recording.
(DOCX)

S2 Table Descriptive statistics of all COP parameters including rm-ANOVA Mauchly’s test of sphericity data. MLD_%: mediolateral displacement; CCD_%: craniocaudal displacement; L_%: length of the COP; AS: average speed of the COP; SS_%: support surface; Angry: hearing angry human voice recording; Happy: hearing happy human voice recording; rm-ANOVA: repeated measure ANOVA, reporting Mauchly’ test of sphericity data (Mauchly’s W, Chi-Square, p-value); %: denotes parameters that were normalized to allow comparison across individuals based on their BOS data in each trial; 95% CI: 95% Confidence Interval, lower (Low) and upper (Up) values are reported;*: Greenhouse–Geisser correction (ε = 0.739) applied; #: Greenhouse–Geisser correction (ε = 0.674) applied.
(DOCX)

S3 Fig A-E. A: ΔCCD_% B: ΔMLD_% C: ΔL_% D: ΔAS E: ΔSS_%. Boxplots depicting the mean, interquartile range, and minimum and maximum values for all 5 evaluated COP parameters. Sound condition (angry, happy) and cluster number (1,2) are presented separately. Outliers are indicated by a circle. MLD_%: mediolateral displacement; CCD_%: craniocaudal displacement; L_%: length of the COP; AS_%: average speed of the COP; SS_%: support surface; Δ: individual dog reaction expressed as percent difference when compared to the no sound condition; cluster_no.: cluster number of each dog based on cluster analysis; Happy: hearing happy human voice recording; Angry: hearing angry human voice recording.
(DOCX)

S4 Table All data used for statistical analysis including abbreviations. (XLSM)
