# Supplementary material for: Effects of happy and angry human voice recordings on postural stability in dogs: An exploratory biomechanical analysis
Source: PLoS One. 2026 Jan 28;21(1):e0339979. doi: 10.1371/journal.pone.0339979 (PMC12851459; doi:10.1371/journal.pone.0339979)
Supplement: S1 Table — MLD_%: mediolateral displacement; CCD_%: craniocaudal displacement; L_%: length of the COP; AS: average speed of the COP; SS_%: support surface; Δ: individual dog reaction expressed as percent difference when compared to the no_sound condition for every COP parameter; cluster: cluster number of each dog based on cluster analysis; Happy: hearing happy human voice recording; Angry: hearing angry human voice recording. (DOCX) [file pone.0339979.s001.docx]

**Table S1.** **Descriptive statistics of all COP parameters and cluster number in different sound conditions per individual dog.**

| dog | condition | MLD % | CCD % | L % | AS | SS % | ΔMLD % | ΔCCD % | ΔL % | ΔAS | ΔSS % | cluster |
| --- | --- | --- | --- | --- | --- | --- | --- | --- | --- | --- | --- | --- |
| Lilo | No sound | 1,22 | 1,34 | 0,10 | 16,34 | 0,08 |  |  |  |  |  |  |
|  | Happy | 1,25 | 0,94 | 0,08 | 18,90 | 0,07 | 2,5 | -29,9 | -20,0 | 15,7 | -12,5 | 2 |
|  | Angry | 1,33 | 0,89 | 0,07 | 20,33 | 0,07 | 9,0 | -33,6 | -30,0 | 24,4 | -12,5 | 2 |
| Finn | No sound | 0,72 | 0,63 | 0,05 | 11,53 | 0,03 |  |  |  |  |  |  |
|  | Happy | 1,23 | 0,99 | 0,07 | 13,06 | 0,07 | 70,8 | 57,1 | 40,0 | 13,3 | 133,3 | 1 |
|  | Angry | 1,29 | 0,97 | 0,07 | 13,39 | 0,08 | 79,2 | 54,0 | 40,0 | 16,1 | 166,7 | 1 |
| Neo | No sound | 0,73 | 0,86 | 0,07 | 17,51 | 0,04 |  |  |  |  |  |  |
|  | Happy | 1,37 | 1,49 | 0,11 | 29,31 | 0,10 | 87,7 | 73,3 | 57,1 | 67,4 | 150,0 | 1 |
|  | Angry | 1,32 | 1,46 | 0,12 | 33,75 | 0,10 | 80,8 | 69,8 | 71,4 | 92,7 | 150,0 | 1 |
| Frida | No sound | 1,06 | 1,28 | 0,07 | 17,10 | 0,05 |  |  |  |  |  |  |
|  | Happy | 1,14 | 1,45 | 0,09 | 20,84 | 0,10 | 7,5 | 13,3 | 28,6 | 21,9 | 100,0 | 1 |
|  | Angry | 1,41 | 1,41 | 0,09 | 22,12 | 0,10 | 33,0 | 10,2 | 28,6 | 29,4 | 100,0 | 1 |
| Diya | No sound | 1,13 | 1,03 | 0,09 | 17,15 | 0,06 |  |  |  |  |  |  |
|  | Happy | 1,31 | 1,29 | 0,08 | 16,08 | 0,11 | 15,9 | 25,2 | -11,1 | -6,2 | 83,3 | 1 |
|  | Angry | 1,24 | 1,42 | 0,09 | 18,30 | 0,10 | 9,7 | 37,9 | 0,0 | 6,7 | 66,7 | 2 |
| Jerry | No sound | 1,00 | 0,67 | 0,07 | 11,55 | 0,05 |  |  |  |  |  |  |
|  | Happy | 1,80 | 1,12 | 0,06 | 13,34 | 0,11 | 80,0 | 67,2 | -14,3 | 15,5 | 120,0 | 1 |
|  | Angry | 1,38 | 0,80 | 0,07 | 14,69 | 0,06 | 38,0 | 19,4 | 0,0 | 27,2 | 20,0 | 2 |
| Ferris | No sound | 1,20 | 1,42 | 0,12 | 31,49 | 0,10 |  |  |  |  |  |  |
|  | Happy | 1,08 | 1,42 | 0,08 | 18,97 | 0,08 | -10,0 | 0,0 | -33,3 | -39,8 | -20,0 | 2 |
|  | Angry | 1,12 | 1,77 | 0,07 | 16,79 | 0,15 | -6,7 | 24,6 | -41,7 | -46,7 | 50,0 | 2 |
| Blizzard | No sound | 1,37 | 1,13 | 0,09 | 19,81 | 0,11 |  |  |  |  |  |  |
|  | Happy | 1,56 | 1,18 | 0,21 | 43,02 | 0,14 | 13,9 | 4,4 | 133,3 | 117,2 | 27,3 | 1 |
|  | Angry | 2,13 | 1,45 | 0,22 | 48,67 | 0,23 | 55,5 | 28,3 | 144,4 | 145,7 | 109,1 | 1 |
| Navy | No sound | 1,08 | 1,02 | 0,07 | 14,45 | 0,07 |  |  |  |  |  |  |
|  | Happy | 1,31 | 1,05 | 0,06 | 17,65 | 0,07 | 21,3 | 2,9 | -14,3 | 22,1 | 0,0 | 2 |
|  | Angry | 1,21 | 0,97 | 0,07 | 17,38 | 0,06 | 12,0 | -4,9 | 0,0 | 20,3 | -14,3 | 2 |
| Kiwi | No sound | 1,29 | 1,28 | 0,10 | 16,56 | 0,09 |  |  |  |  |  |  |
|  | Happy | 0,86 | 1,84 | 0,07 | 12,30 | 0,10 | -33,3 | 43,8 | -30,0 | -25,7 | 11,1 | 2 |
|  | Angry | 1,52 | 1,44 | 0,08 | 12,50 | 0,16 | 17,8 | 12,5 | -20,0 | -24,5 | 77,8 | 2 |
| Nori | No sound | 1,56 | 1,32 | 0,10 | 22,19 | 0,08 |  |  |  |  |  |  |
|  | Happy | 1,32 | 1,38 | 0,13 | 30,70 | 0,14 | -15,4 | 4,5 | 30,0 | 38,4 | 75,0 | 1 |
|  | Angry | 1,13 | 1,40 | 0,18 | 35,44 | 0,10 | -27,6 | 6,1 | 80,0 | 59,7 | 25,0 | 2 |
| Grisu | No sound | 1,27 | 1,35 | 0,06 | 15,70 | 0,10 |  |  |  |  |  |  |
|  | Happy | 0,93 | 0,97 | 0,07 | 17,35 | 0,05 | -26,8 | -28,1 | 16,7 | 10,5 | -50,0 | 2 |
|  | Angry | 0,83 | 0,81 | 0,06 | 14,48 | 0,04 | -34,6 | -40,0 | 0,0 | -7,8 | -60,0 | 2 |
| Jade | No sound | 1,59 | 1,04 | 0,18 | 23,17 | 0,10 |  |  |  |  |  |  |
|  | Happy | 1,70 | 1,28 | 0,12 | 13,28 | 0,12 | 6,9 | 23,1 | -33,3 | -42,7 | 20,0 | 2 |
|  | Angry | 1,98 | 1,23 | 0,13 | 19,42 | 0,13 | 24,5 | 18,3 | -27,8 | -16,2 | 30,0 | 2 |
| Semmel | No sound | 1,82 | 1,52 | 0,11 | 19,58 | 0,18 |  |  |  |  |  |  |
|  | Happy | 1,36 | 1,30 | 0,09 | 27,48 | 0,09 | -25,3 | -14,5 | -18,2 | 40,3 | -50,0 | 2 |
|  | Angry | 1,47 | 1,16 | 0,09 | 23,49 | 0,09 | -19,2 | -23,7 | -18,2 | 20,0 | -50,0 | 2 |
| Filou | No sound | 1,71 | 1,47 | 0,26 | 38,02 | 0,12 |  |  |  |  |  |  |
|  | Happy | 2,20 | 2,11 | 0,28 | 45,01 | 0,27 | 28,7 | 43,5 | 7,7 | 18,4 | 125,0 | 1 |
|  | Angry | 2,05 | 1,39 | 0,18 | 29,60 | 0,16 | 19,9 | -5,4 | -30,8 | -22,1 | 33,3 | 2 |
| Lola | No sound | 1,98 | 1,19 | 0,16 | 33,59 | 0,12 |  |  |  |  |  |  |
|  | Happy | 1,91 | 0,77 | 0,05 | 11,77 | 0,09 | -3,5 | -35,3 | -68,8 | -65,0 | -25,0 | 2 |
|  | Angry | 1,23 | 1,03 | 0,06 | 12,83 | 0,07 | -37,9 | -13,4 | -62,5 | -61,8 | -41,7 | 2 |
| Manfred | No sound | 1,26 | 1,37 | 0,15 | 31,47 | 0,10 |  |  |  |  |  |  |
|  | Happy | 2,46 | 1,25 | 0,15 | 29,45 | 0,29 | 95,2 | -8,8 | 0,0 | -6,4 | 190,0 | 1 |
|  | Angry | 1,56 | 1,36 | 0,14 | 26,45 | 0,13 | 23,8 | -0,7 | -6,7 | -16,0 | 30,0 | 2 |
| Desper | No sound | 1,54 | 1,67 | 0,08 | 16,15 | 0,09 |  |  |  |  |  |  |
|  | Happy | 1,10 | 0,99 | 0,07 | 16,38 | 0,06 | -28,6 | -40,7 | -12,5 | 1,4 | -33,3 | 2 |
|  | Angry | 1,13 | 1,46 | 0,09 | 21,38 | 0,09 | -26,6 | -12,6 | 12,5 | 32,4 | 0,0 | 2 |
| Hiska | No sound | 1,04 | 1,22 | 0,07 | 13,87 | 0,07 |  |  |  |  |  |  |
|  | Happy | 1,70 | 1,40 | 0,11 | 17,54 | 0,09 | 63,5 | 14,8 | 57,1 | 26,5 | 28,6 | 1 |
|  | Angry | 2,52 | 1,67 | 0,18 | 26,07 | 0,23 | 142,3 | 36,9 | 157,1 | 88,0 | 228,6 | 1 |
| Uno | No sound | 1,40 | 1,03 | 0,07 | 21,01 | 0,08 |  |  |  |  |  |  |
|  | Happy | 2,10 | 1,12 | 0,09 | 16,42 | 0,15 | 50,0 | 8,7 | 28,6 | -21,8 | 87,5 | 1 |
|  | Angry | 2,23 | 0,97 | 0,08 | 16,79 | 0,17 | 59,3 | -5,8 | 14,3 | -20,1 | 112,5 | 2 |
| Quentin | No sound | 0,90 | 0,91 | 0,09 | 24,52 | 0,06 |  |  |  |  |  |  |
|  | Happy | 1,61 | 1,16 | 0,12 | 30,63 | 0,09 | 78,9 | 27,5 | 33,3 | 24,9 | 50,0 | 1 |
|  | Angry | 1,92 | 1,79 | 0,11 | 26,45 | 0,16 | 113,3 | 96,7 | 22,2 | 7,9 | 166,7 | 1 |
| Miley | No sound | 1,38 | 1,03 | 0,13 | 26,19 | 0,08 |  |  |  |  |  |  |
|  | Happy | 1,48 | 1,10 | 0,14 | 29,67 | 0,09 | 7,2 | 6,8 | 7,7 | 13,3 | 12,5 | 2 |
|  | Angry | 1,93 | 1,52 | 0,12 | 29,66 | 0,21 | 39,9 | 47,6 | -7,7 | 13,2 | 162,5 | 1 |
| Amadeus | No sound | 1,03 | 1,03 | 0,12 | 21,26 | 0,05 |  |  |  |  |  |  |
|  | Happy | 1,65 | 1,24 | 0,12 | 16,73 | 0,09 | 60,2 | 20,4 | 0,0 | -21,3 | 80,0 | 1 |
|  | Angry | 1,35 | 0,87 | 0,11 | 18,11 | 0,06 | 31,1 | -15,5 | -8,3 | -14,8 | 20,0 | 2 |

MLD_%: mediolateral displacement; CCD %: craniocaudal displacement; L_%: length of the COP; AS: average speed of the COP; SS_%: support surface; Δ: individual dog reaction expressed as percent difference when compared to the no sound condition for every COP parameter; cluster: cluster number of each dog based on cluster analysis; Happy: hearing happy human voice recording; Angry: hearing angry human voice recording; %: denotes parameters that were normalized to allow comparison across individuals based on their BOS data in each trial.
