# Supplementary material for: Effects of happy and angry human voice recordings on postural stability in dogs: An exploratory biomechanical analysis
Source: PLoS One. 2026 Jan 28;21(1):e0339979. doi: 10.1371/journal.pone.0339979 (PMC12851459; doi:10.1371/journal.pone.0339979)
Supplement: S2 Table — MLD_%: mediolateral displacement; CCD_%: craniocaudal displacement; L_%: length of the COP; AS: average speed of the COP; SS_%: support surface; Angry: hearing angry human voice recording; Happy: hearing happy human voice recording; rm-ANOVA: repeated measure ANOVA, reporting Mauchly’ test of sphericity data (Mauchly’s W, Chi-Square, p-value); %: denotes parameters that were normalized to allow comparison across individuals based on their BOS data in each trial; 95% CI: 95% Confidence Interval, lower (Low) and upper (Up) values are reported;*: Greenhouse–Geisser correction (ε = 0.739) applied; #: Greenhouse–Geisser correction (ε = 0.674) applied. (DOCX) [file pone.0339979.s002.docx]

**Table S2. Descriptive statistics of all COP parameters including rm-ANOVA Mauchly’s test of sphericity data.**

|  | |  | |  | | |  | |  |  |
| --- | --- | --- | --- | --- | --- | --- | --- | --- | --- | --- |
|  | condition | | mean ± SE | 95 % CI [Low, Up] | Mauchly's W | Chi-Square | | p-value |  |  |
| MLD_% | Angry | | 1.54 ± 0.09 | [1.35, 1.72] |  |  | |  |  |  |
|  | Happy | | 1.50 ± 0.09 | [1.32, 1.67] |  |  | |  |  |  |
|  | No- sound | | 1.27 ± 0.07 | [1.13, 1.41] |  |  | |  |  |  |
|  | rm-ANOVA | |  |  | 0.91 | 2.05 | | 0.36 |  |  |
| CCD_% | Angry | | 1.27 ± 0.06 | [1.14, 1.40] |  |  | |  |  |  |
|  | Happy | | 1.25 ± 0.06 | [1.13, 1.38] |  |  | |  |  |  |
|  | No- sound | | 1.17 ± 0.05 | [1.05, 1.28] |  |  | |  |  |  |
|  | rm-ANOVA | |  |  | 0.98 | 0.35 | | 0.84 |  |  |
| L_% | Angry | | 0.11 ± 0.01 | [0.09, 0.13] |  |  | |  |  |  |
|  | Happy | | 0.11 ± 0.01 | [0.08, 0.13] |  |  | |  |  |  |
|  | No- sound | | 0.11 ± 0.01 | [0.08, 0.13] |  |  | |  |  |  |
|  | rm-ANOVA | |  |  | 0.65 | 9.17 | | 0.01* |  |  |
| AS | Angry | | 22.53 ± 1.82 | [18.76, 26.30] |  | | |  |  |  |
|  | Happy | | 21.99 ± 1.97 | [17.92, 26.08] |  | | |  |  |  |
|  | No- sound | | 20.88 ± 1.49 | [17.79, 23.97] |  | | |  |  |  |
|  | rm-ANOVA | |  |  | 0.52 | 13.86 | | <0.01^#^ |  |  |
| SS_% | Angry | | 0.12 ± 0.01 | [0.09, 0.14] |  |  | |  |  |  |
|  | Happy | | 0.11 ± 0.01 | [0.09, 0.14] |  |  | |  |  |  |
|  | No- sound | | 0.08 ± 0.01 | [0.07, 0.10] |  |  | |  |  |  |
|  | rm-ANOVA | |  |  | 0.98 | 0.41 | | 0.82 |  |  |
|  | | | | |  |  | |  |  |  |
|  | | | | |  |  | |  |  |  |

MLD_%: mediolateral displacement; CCD %: craniocaudal displacement; L_%: length of the COP; AS: average speed of the COP; SS_%: support surface; Angry: hearing angry human voice recording; Happy: hearing happy human voice recording; rm-ANOVA: repeated measure ANOVA, reporting Mauchly’ test of sphericity data (Mauchly’s W, Chi-Square, p-value); %: denotes parameters that were normalized to allow comparison across individuals based on their BOS data in each trial; 95% CI: 95% Confidence Interval, lower (Low) and upper (Up) values are reported;*: Greenhouse–Geisser correction (ε = 0.739) applied; #: Greenhouse–Geisser correction (ε = 0.674) applied.
