# Supplementary material for: Effects of happy and angry human voice recordings on postural stability in dogs: An exploratory biomechanical analysis
Source: PLoS One. 2026 Jan 28;21(1):e0339979. doi: 10.1371/journal.pone.0339979 (PMC12851459; doi:10.1371/journal.pone.0339979)
Supplement: S3 Fig — Sound condition (angry, happy) and cluster number (1,2) are presented separately. Outliers are indicated by a circle. MLD_%: mediolateral displacement; CCD_%: craniocaudal displacement; L_%: length of the COP; AS_%: average speed of the COP; SS_%: support surface; Δ: individual dog reaction expressed as percent difference when compared to the no sound condition; cluster_no.: cluster number of each dog based on cluster analysis; Happy: hearing happy human voice recording; Angry: hearing angry human voice recording. (DOCX) [file pone.0339979.s003.docx]

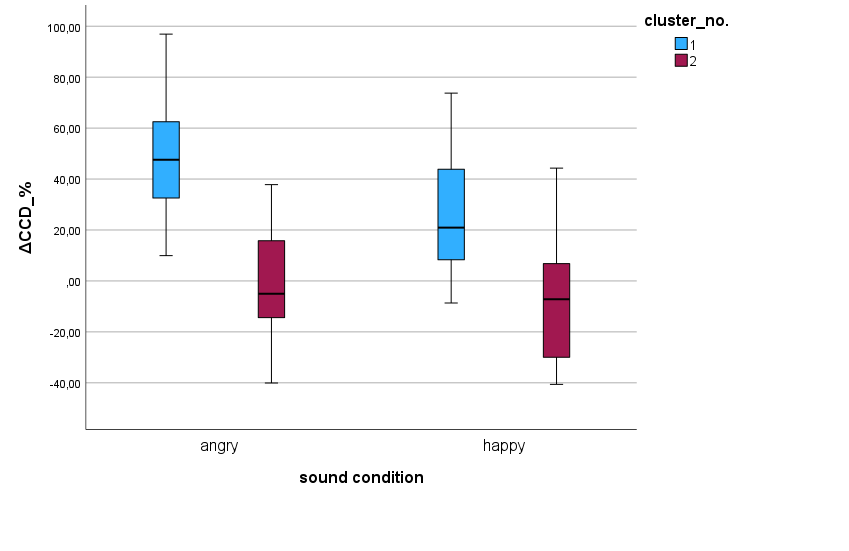


Fig. S3_A: ΔCCD_%


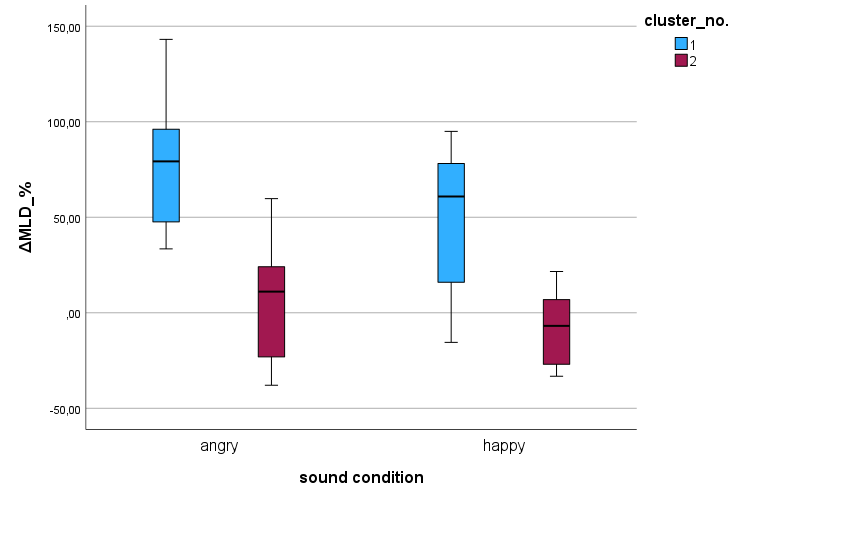


Fig. S3_B: ΔMLD_%


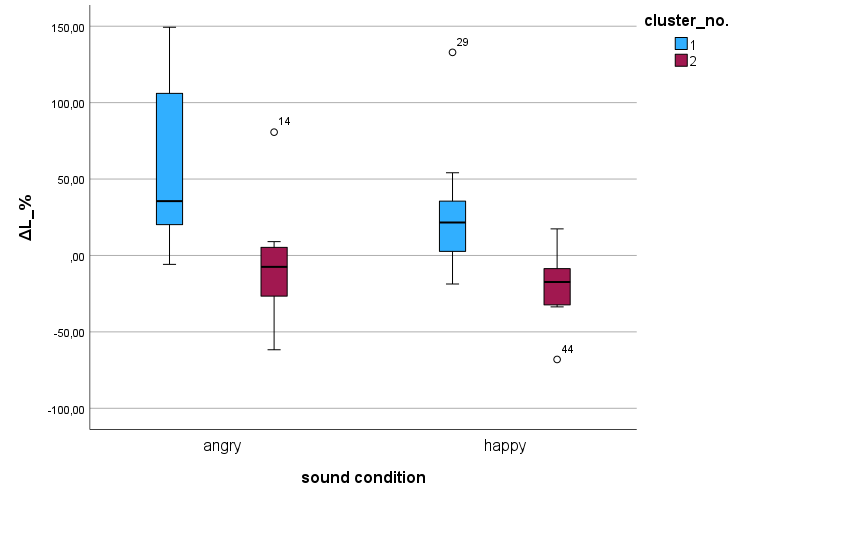


Fig. S3_C: ΔL_%


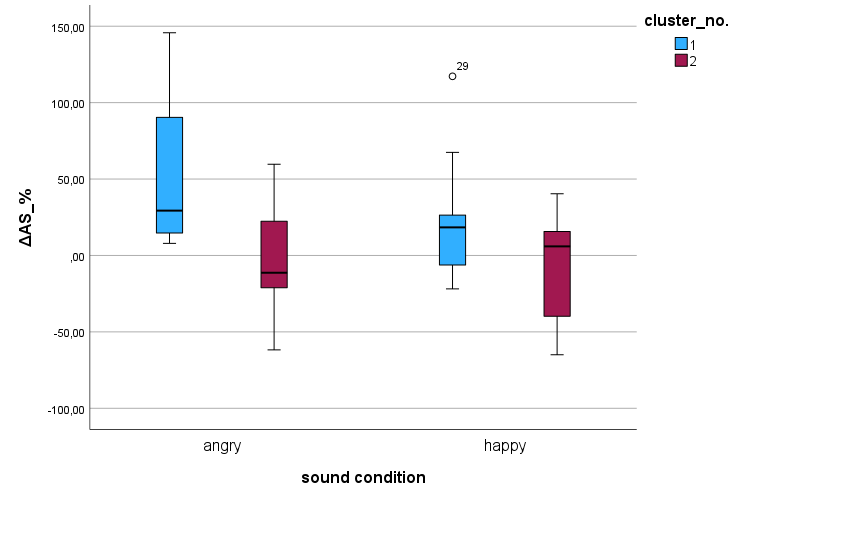


Fig_S3_D: Δ AS


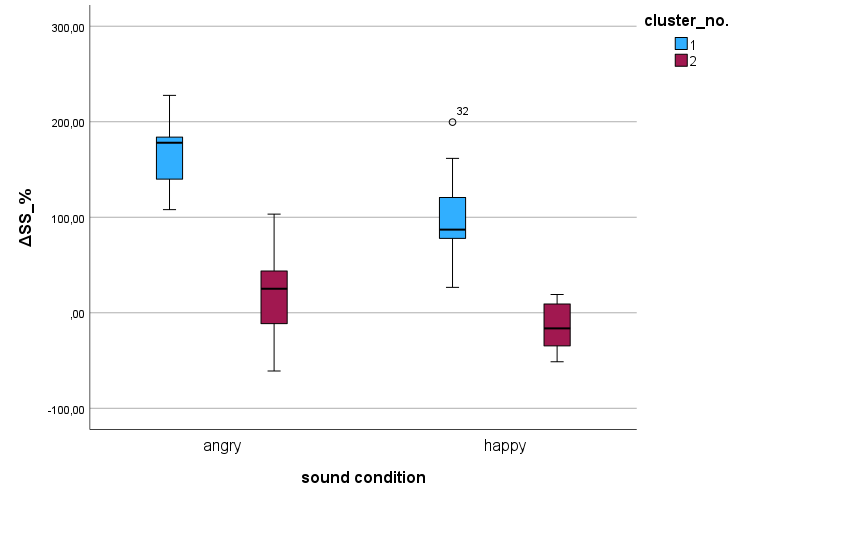


Fig. S3_E: ΔSS_%

**Figure S3 A-E: Boxplots depicting the mean, interquartile range, and minimum and maximum values for all 5 evaluated COP parameters.** Sound condition (angry, happy) and cluster number (1,2) are presented separately. Outliers are indicated by a circle. MLD_%: mediolateral displacement; CCD_%: craniocaudal displacement; L_%: length of the COP; AS_%: average speed of the COP; SS_%: support surface; Δ: individual dog reaction expressed as percent difference when compared to the no sound condition; %: denotes parameters that were normalized to allow comparison across individuals based on their BOS data in each trial; cluster_no.: cluster number of each dog based on cluster analysis; Happy: hearing happy human voice recording; Angry: hearing angry human voice recording;
